# Exogenous *p*-Coumaric Acid Improves *Salvia hispanica* L. Seedling Shoot Growth

**DOI:** 10.3390/plants8120546

**Published:** 2019-11-26

**Authors:** Mbukeni Nkomo, Arun Gokul, Marshall Keyster, Ashwil Klein

**Affiliations:** 1 Plant Omics Laboratory, Department of Biotechnology, University of the Western Cape, Bellville 7535, South Africa; 2870274@myuwc.ac.za; 2 Environmental Biotechnology Laboratory, Department of Biotechnology, University of the Western Cape, Bellville 7535, South Africa; 3107408@myuwc.ac.za; 3 DST-NRF Centre of Excellence in Food Security, University of the Western Cape, Bellville 7530, South Africa

**Keywords:** chia, chlorophyll content, *p*-coumaric acid, osmoprotectants, oxidative stress, proline, superoxide dismutase

## Abstract

*p*-Coumaric acid (*p*-CA) belongs to a family of natural esters of hydroxycinnamic acid compounds that have been shown to modulate plant growth and metabolism. In this study, we investigated the effect of exogenous *p*-CA on plant growth, reactive oxygen species (ROS)-induced oxidative damage, photosynthetic metabolism, osmolyte content and changes in superoxide dismutase (SOD) enzymatic activity. Exogenous *p*-CA improved *Salvia hispanica* (chia) growth by significantly enhancing shoot length, fresh and dry weights coupled with augmented levels of total chlorophyll and carotenoid contents. Furthermore, *p*-CA also triggered an induction in proline, glycine betaine (GB) and superoxide (O_2_**^∙^**^−^) levels while no changes were observed for hydrogen peroxide (H_2_O_2_) and downstream malondialdehyde (MDA) content. Also, no change in SOD activity was observed in the *p*-CA treatment relative to the control. Therefore, the results suggest that exogenous *p*-CA improves chia seedling growth possibly via activation of a ROS-signalling pathway involving O_2_**^∙^**^−^ under the control of proline accumulation.

## 1. Introduction

Phenolic acids are divided into two groups based on their chemical structure, namely hydroxybenzoic and hydroxycinnamic [1]. Hydroxycinnamic acids (includes *p*-coumaric-, caffeic-, ferulic-, and sinapic acid) derived from the phenylalanine pathway, have gained recent attention due to their revealed properties, such as antimicrobial, antitumor, anti-inflammatory, antioxidant and other health benefits like antidiabetic activity [2,3,4]. In plants, these hydroxycinnamic acids have been referred to as allelochemicals and are commonly found in soils at concentrations ranging from 0.01 to 0.1 mM and have been shown to affect plant growth at concentrations of up to 10 mM [5,6]. Of these hydroxycinnamic acids, *p*-CA resides at a metabolically important position, linked to the synthesis of other hydroxycinnamic acids such as caffeic-, ferulic- and sinapic acid. A few lines of research have shown that *p*-CA reduced the rate of seed germination, root length and biomass in different plant species [7,8,9,10,11]. This led to a general perception that exogenously applied *p*-CA restricts plant growth and development in various plant species [7,8,9,10,11,12,13]. Jones et al. [14] showed that exogenous application of caffeic acid (a derivative of *p*-CA) improved the growth of chia plants through differential regulation of photosynthetic metabolism, ROS content and antioxidant enzyme activities under salt stress conditions. This result is in contradiction to what was reported for legume plants [15,16]. Antioxidant enzymes such as SOD, ascorbate peroxidase (APX) and catalase (CAT) differentially regulate ROS biosynthesis. These ROS molecules include O_2_**^∙^**^−^, H_2_O_2_ and hydroxyl radicals (OH**^∙^**) and if not controlled can lead to oxidative stress manifested as enhanced cellular death. However, increased ROS molecules (in moderation)—for example O_2_**^∙^**^−^ and H_2_O_2_—have been shown to act as signalling molecules in various plant species [17,18]. Furthermore, a study by Gokul et al. [19] showed that O_2_**^∙^**^−^ and H_2_O_2_ regulate physio-biochemical responses in *Brassica napus* plants via the modulation of SOD and APX activity, which in turn led to increased seedling growth. This evidence suggests that the mechanism controlling redox homeostasis of ROS molecules can be exploited to improve plant growth. To our knowledge, no study has investigated the effect of exogenous application of *p*-CA on the physio-biochemical responses (including ROS homeostasis) of pseudocereal plants.

Chia is an oilseed pseudocereal plant known for its nutritional and health promoting properties. The seed is a natural source of omega-3 fatty acids (α-linolenic acid), soluble and insoluble fibers, and proteins in addition to other important nutritional components, such as vitamins, minerals, and natural antioxidants [20,21]. Furthermore, it has been shown that chia plants (seeds and leaves) contain various bioactive components such as tocopherols and phenolic compounds, which reduces the risk of liver, cardiovascular and obesity-related diseases [22,23,24,25,26]. In view of the nutritional and health-promoting properties of chia plants in recent years, there has been considerable interest to explore the biological and technological potential of this plant. In this study, we investigated the effect of exogenously applied *p*-CA on the growth, photosynthetic pigments, osmolyte content, ROS-induce oxidative damage and changes in SOD activity in chia seedlings.

## 2. Results

### 2.1. p-CA Improves Chia Seedling Growth

**Figure 1:**
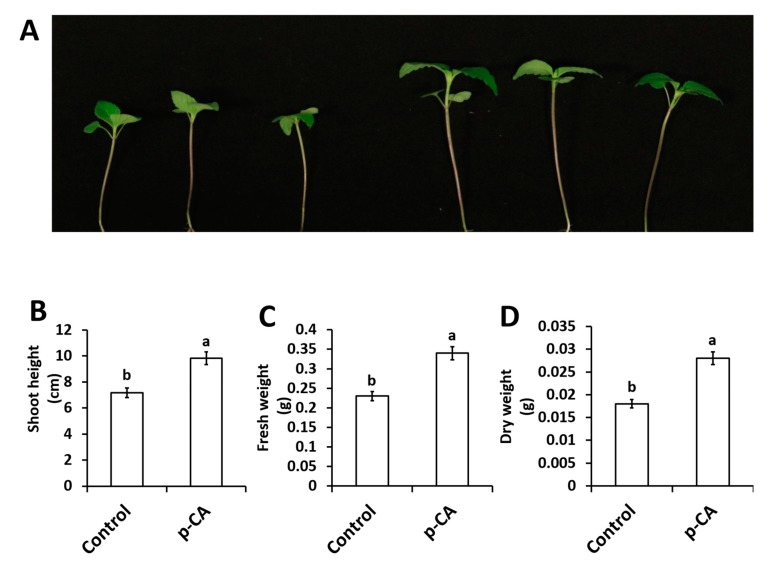
Representative chia seedling shoots under control and *p*-CA treatments (**A**). Shoot height (**B**), shoot fresh weight (**C**) and shoot dry weight (**D**) of chia seedlings treated with *p*-CA. Data represent the mean (± SE) from six independent experiments. Different letters represent statistical significance at *p* < 0.05 (Tukey–Kramer test).

In this study we investigated the effect of exogenous application of *p*-CA on plant growth and chlorophyll content. The results show that exogenous *p*-CA significantly increase shoot growth of chia seedlings (Figure 1), as observed for shoot height (SH), fresh weight (FW) and dry weight (DW). Exogenous *p*-CA improved SH by 37.2% when compared to control plants (Figure 1B). A similar trend was observed for FW and DW. On average, FW and DW were enhanced by 43.6% and 54.1%, respectively, under *p*-CA treatment relative to the control (Figure 1C,D).

### 2.2. The Effect of Exogenous p-CA on Chlorophyll Metabolism and Osmolyte Content

Both chlorophyll *a* and chlorophyll *b* content showed similar results that significantly increased by 36.2% and 38.5%, respectively, when compared to the control. Parallel to chlorophyll *a* and chlorophyll *b*, there was a significant increase in total chlorophyll (37.6%) and carotenoid (25.1%) content in response to exogenous application of *p*-CA (Table 1). The *p*-CA treatment also caused a significant increase in osmolyte compounds (GB and proline), when compared to the control. Furthermore, there was a significant increase in both proline (170%) and GB (22.4%) content (Table 1) when compared to the control.

### 2.3. Effects of p-CA on Superoxide Radical and Superoxide Dismutase Activity

**Figure 2:**
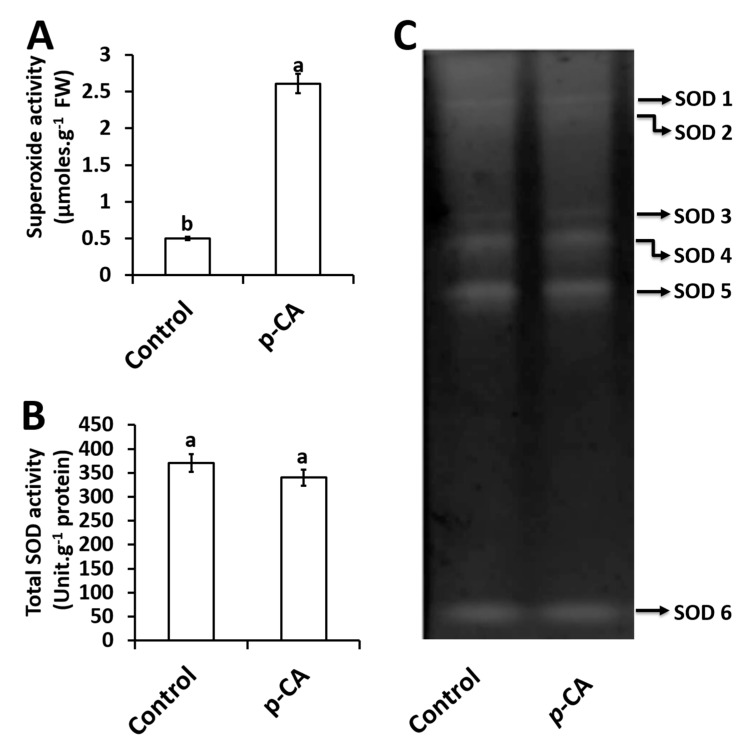
Superoxide content (**A**), total SOD activity (**B**) and the activity of individual SOD isoforms (**C**) in control and *p*-CA treated chia seedlings. Data represent the mean (± SE) from six independent experiments. Different letters represent statistical significance at *p* < 0.05 (Tukey–Kramer test).

Exogenous application of *p*-CA significantly increased O_2_**^∙^**^−^ content by 522% relative to the control (Figure 2A).

For total SOD activity, no significant changes were observed in response to exogenous *p*-CA when compared to the control (Figure 2B). A total of six SOD isoforms were detected and named SOD1–6 (Figure 2C). Upon visual inspection together with densitometry analysis (data not shown) no significant difference was observed for all 6 isoforms in the *p*-CA treatment when compared to the control (Figure 2C).

### 2.4. Effects of Exogenous Application of p-CA on H_2_O_2_ Content and the Extent of Lipid Peroxidation

**Figure 3:**
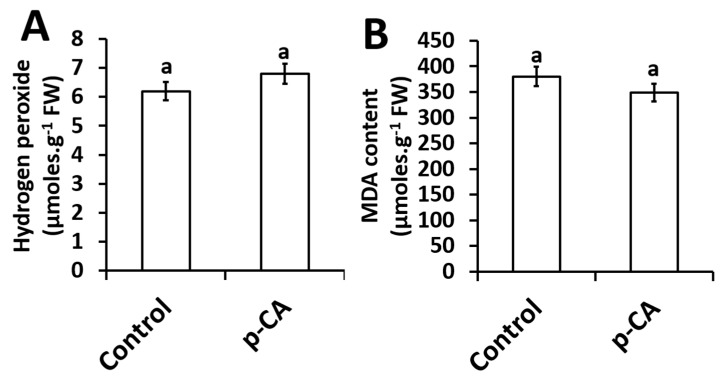
Hydrogen peroxide content (**A**) and MDA content (**B**) in chia seedling shoots under control and *p*-CA treatment. Data represent the mean (± SE) six independent experiments. Different letters represent statistical significance at *p* < 0.05 (Tukey–Kramer test).

The effect of exogenous *p*-CA on H_2_O_2_ content and MDA content (an indicator of lipid peroxidation) in the shoots of chia seedlings were investigated. Compared to the control, *p*-CA had no effect on H_2_O_2_ content (Figure 3A). Based on the results presented here, no significant changes in the extent of lipid peroxidation was observed in shoots of chia seedlings treated with *p*-CA (Figure 3B).

## 3. Discussion

In this study, we have dissected the role of exogenous *p*-CA on chia plant growth and biochemistry. Exogenous application of *p*-CA has been studied previously in various plant species [7,9,13,27]. Reigosa and colleagues [11] performed a germination study on six weed species using different phenolic compounds including *p*-CA and observed that higher concentrations of *p*-CA inhibited the rate of germination while low concentrations had no significant effect on all six weed species. That result is in agreement with our preliminary investigation conducted on chia seeds on the rate of germination under different concentrations of *p*-CA (0 μM, 100 μM, 250 μM, 500 μM, and 1000 μM). We observed that the final seed germination percentage was similar across all treatments except for 1000 μM at which no germination occurred (see Supplementary Data; Figure S1B). Our data also showed no effect on sprout growth across all treatments with the exception at 500 μM *p*-CA (see Supplementary Data; Figure S1A). Furthermore, we identified 100 μM as a suitable concentration for further studies on chia seedling growth, physiology and biochemistry. Previous work on *p*-CA supplementation demonstrated that treatment with 100 μM *p*-CA reduced cucumber leaves [28]. Similar results were also observed in other studies using various concentrations (100 μM–1000 μM) of *p*-CA. In those studies, it was demonstrated that exogenous application of *p*-CA had inhibitory effects on root length, root fresh and dry weights of many tested plant species [7,8,9,10,13,27]. Contrastingly, an opposite phenomenon was observed in our study because we observed that treatment with 100 μM *p*-CA promoted chia seedling growth and biomass (Figure 1). In addition, we examined chlorophylls *a* and *b*, total chlorophyll and carotenoid contents in the shoots of chia seedlings treated with *p*-CA compared to untreated controls. We observed an increase in chlorophyll pigments (Table 1) in chia seedlings treated with *p*-CA, which suggests that exogenous *p*-CA positively regulates chlorophyll synthesis in chia plants. Therefore, we hypothesized that the increase in growth could be as a result of an increase in chlorophyll pigments. This hypothesis is supported by Yan et al. [29] that observed a positive correlation between chlorophyll content and biomass. Contrasting results were observed by [30], which treated soybean (*Glycine max*) seedlings with exogenous *p*-CA and observed a reduction in chlorophyll content. Einhellig and colleagues [30] suggested that the decrease in chlorophyll content in soybean might be as a result of other secondary responses, as the same phenomenon was not observed in sorghum seedlings. These secondary responses include ROS molecules such as O_2_**^∙^**^−^, which have been shown to interact directly with chlorophyll *a* ultimately leading to chlorophyll degradation [31], however Yan et al. [29] observed an increase in chlorophyll content and O_2_**^∙^**^−^ content in response to exogenous salicylic acid and sodium nitroprusside (nitric oxide donor) in wheat. In contrast, exogenous caffeic acid decreased O_2_**^∙^**^−^ content in soybean plants [32], which led to unaltered levels of chlorophyll content [16]. These studies point to a complex interaction between O_2_**^∙^**^−^ and chlorophyll in different plants, which has not been elucidated to date. In our study, higher levels of O_2_**^∙^**^−^ were detected under exogenous *p*-CA and the scavenging mechanism of O_2_**^∙^**^−^ did not involve a direct role of SOD enzymes. We observed that total SOD activity and individual SOD isoforms were unaltered in *p*-CA treated chia seedlings, which suggests that other mechanisms of O_2_**^∙^**^−^ scavenging were possibly triggered by exogenous *p*-CA in the chia seedlings. This phenomenon was also observed by Yan et al. [29], where the increase in O_2_**^∙^**^−^ did not result in an increase in SOD activity in response to exogenous sodium nitroprusside. Contrastingly, Klein et al. [32] showed that exogenous caffeic acid decreased O_2_**^∙^**^−^ content in soybean plants whereas a significant increase in SOD activity was observed. These studies show a complex interaction between O_2_**^∙^**^−^ accumulation and scavenging via SOD, which is not well understood.

We also observed that H_2_O_2_ levels were similar to those of the control seedlings. This finding supports our result where no changes in SOD activity were observed. According to the Asada–Halliwell pathway, SOD scavenges O_2_**^∙^**^−^ and produces downstream H_2_O_2_, which has the ability to oxidize polyunsaturated fatty acids (PUFA) ultimately producing secondary products such as MDA, which is an indicator of lipid peroxidation [33]. Lipid peroxidation is a marker for testing membrane cellular damage following oxidative stress [34]. In our study, we observed unaltered levels of MDA in *p*-CA treated seedlings when compared to the controls (Figure 3B), which might be expected considering no changes in H_2_O_2_ content were observed under *p*-CA supplementation.

The protective effects of compatible osmolytes (GB and proline) in limiting membrane injury have been reported and studies have shown that proline can scavenge ROS molecules [35,36]. Furthermore, compatible osmolytes can also act as osmoprotectants of cellular molecules under a wide range of abiotic stresses [37,38,39,40]. Matysik and colleagues [41] showed that proline can protect the photosystem PSII by scavenging O_2_**^∙^**^−^ thus reducing lipid peroxidation in the thylakoid membranes. However, our study is in agreement with the results obtained by [14], which showed that exogenous supplementation of hydroxycinnamic acids (caffeic acid) increased chia plant growth. We propose a mechanism by which exogenous application of *p*-CA improves the growth of chia shoots, possibly through the activation of O_2_**^∙^**^−^. This is indicated by our experiments where both H_2_O_2_ and total SOD activity were not affected (Figure 3). In support of our results, there are several reports showing that, besides directly scavenging O_2_**^∙^**^−^ [42], *p*-CA can also increase proline accumulation, which has also been linked to O_2_**^∙^**^−^ scavenging [26,35].

## 4. Materials and Methods

### 4.1. Plant Material and Growth Conditions

Chia seeds purchased from Faithful to Nature, Cape Town, South Africa were used in this experiment. The seeds were germinated on wet filter paper in the dark for a period of 72 h. Germinated seeds were transferred to 19/20 cm plastic pots containing moist promix growth medium (Stodels Garden Centre, Brackenfell, South Africa) and allowed to grow on a 27/19 °C day/night temperature cycle under a 16/8 h dark cycle at a photosynthetic photon flux density of 300 μmol photons·m^−2^·s^−1^ during the day phase until the end of the experiment. Seedlings were grown in a completely randomized design to eliminate the effect of variations in environmental conditions at different positions in the growth room.

Plants at the same developmental stage and of similar height were selected for all experiments. Control plants were supplemented with 50 mL of Nitrosol^(R)^ solution diluted in water (1:300). For treatment with *p*-CA, plants were supplemented with Nitrosol^(R)^ containing 100 µM *p*-CA (at 2-day intervals) for a period of 14 days.

### 4.2. Measurement of Plant Growth

Chia seedlings were carefully removed from the growth medium to avoid damage. Subsequently, the roots were separated from the shoots to prevent erroneous data interpretation caused by possible root damage. The shoots from each treatment were scored for length (SL), fresh weight (FW) and dry weight (DW). The DW was determined by drying the seedlings in an oven at 55 °C for 48 h as described by [19].

### 4.3. Chlorophyll Estimation

Total chlorophyll content in the shoots of chia seedlings was estimated using a method previously described by [43]. Freshly harvested shoots (200 mg per treatment) was homogenized with 5 mL of dimethylsulfoxide (DMSO) and incubated at 65 °C for 3 h. The absorbance rates of the extract (200 μL) were recorded at 645 nm and 663 nm, with DMSO used as a blank using a FLUOstar Omega UV-visible spectrophotometer (BMG LabTech GmbH, Ortenberg, Germany).

### 4.4. Protein Extraction for Biochemical Analysis

Shoots from all treatments were harvested and ground into a fine powder using liquid nitrogen. Shoots (0.1 g) was homogenized in 1 mL of PVP buffer (40 mM K_2_HPO_4_ at pH 7.4; 1 mM EDTA; 5% PVP MW = 40 000; 5% glycerol in distilled H_2_O) for the measurement and detection of SOD enzymatic activity. Protein concentrations were determined using the RC DC Protein Assay Kit 11 (Bio-Rad Laboratories).

### 4.5. Assays for ROS Content

Superoxide (O_2_**^∙^**^−^) content in the shoots of chia seedlings was quantified using a method previously described in [19]. Superoxide concentrations were determined by submerging intact seedling shoots in a solution containing; 10 mM KCN (to inhibit Cu/Zn SODs), 10 mM H_2_O_2_ (to inhibit Mn and Cu/Zn SODs), 2% (*w*/*v*) SDS (to inhibit Mn and Fe SODs), 80 mM Nitroblue tetrazolium chloride (NBT) and 50 mM potassium phosphate (pH 7.0) and incubated for 20 min. After incubation, shoots were homogenized and centrifuged at 10 000 × g for 5 min. The resulting supernatant was spectrophotometrically analysed at 600 nm using a FLUOstar Omega UV-visible spectrophotometer (BMG LabTech GmbH, Ortenberg, Germany). The superoxide concentration was calculated using the NBT extinction coefficient of 12.8 mM·cm^−1^. Hydrogen peroxide (H_2_O_2_) content was measured in shoots of chia seedlings, using a method previously described by [44]. Briefly, shoot material (0.1 g) were ground to a fine powder in liquid nitrogen and homogenized in 1 mL of cold 6% (*w*/*v*) TCA. The extracts were centrifuged at 12 000 × g for 30 min at 4 °C and 50 μL of the supernatant was used to initiate the reaction in a mixture (total volume of 200 μL) containing 5 mM K_2_HPO_4_ (pH 5.0) and 0.5 M potassium iodide (KI). The reaction mixture was incubated at 25 °C for 20 min and the absorbance readings were recorded at 390 nm using a FLUOstar Omega UV-visible spectrophotometer (BMG LabTech GmbH, Ortenberg, Germany). H_2_O_2_ content was calculated using a standard curve based on the absorbance of H_2_O_2_ standards.

### 4.6. Determination of MDA Content

The extent of lipid peroxidation (MDA) in the shoots of chia seedlings were quantified as described by [45]. Briefly, shoot material (0.1 g) were ground to a fine powder in liquid nitrogen and homogenized in 1 mL of cold 6% (*w*/*v*) TCA. Aliquots of the supernatant (100 μL), were mixed with 400 μL of 0.5% thiobarbituric acid (TBA; prepared in 20% TCA). The mixture was incubated at 95 °C for 30 min and the reaction was terminated on ice for 5 min. The mixture was centrifuged at 12 000 × g for 5 min at 4 °C. The absorbance of the supernatant was recorded at 532 nm and 600 nm using a FLUOstar Omega UV-visible spectrophotometer (BMG LabTech GmbH, Ortenberg, Germany). After subtracting the non-specific absorbance, the MDA concentration was calculated using the extinction coefficient of 155 mM·cm^−1^.

### 4.7. Quantification of SOD Activity

Total SOD activity was measured using a method previously described by [46]. The protein extract (10 μL) was mixed with 190 μL of the assay buffer (50 mM K_2_HPO_4_, pH 7.8, 0.1 mM EDTA, 10 mM methionine, 5 μM riboflavin, 0.1 mM NBT) and incubated at room temperature for 20 min under fluorescent light. Subsequently, the absorbance readings were recorded at 560 nm using a FLUOstar Omega UV-visible spectrophotometer (BMG LabTech GmbH, Ortenberg, Germany). SOD activity was calculated based on the amount of enzyme that was required to cause a 50% reduction of NBT.

In-gel activity of individual SOD isoforms was detected in the shoots of chia seedlings according to a method described by [32]. Protein extracts (100 μg) from each treatment were separated on a 10% (v/v) resolving native polyacrylamide gel at 4 °C. SOD activity of individual isoforms was detected by photochemical staining with riboflavin and NBT.

### 4.8. Determination of Proline Content

Total free proline content in the shoots of chia plants was estimated using [47] and modified by [48]. Briefly, fresh shoot material from each treatment (0.1 g) were homogenized in 500 µL of 3% (*w*/*v*) sulphosalicylic acid using a mortar and pestle. About 200 µL ml of each homogenate was mixed with 200 µL of glacial acetic acid to which 200 µL of ninhydrin was added. The reaction mixture was boiled in a water bath at 100 °C for 30 min and immediately cooled in an ice bath. After cooling, 400 µL of toluene was added to the reaction mixture. After thorough mixing, the chromophore containing toluene was separated and the absorbance of the red color developed was read at 520 nm against the toluene blank on FLUOstar Omega UV-visible spectrophotometer (BMG LabTech GmbH, Ortenberg, Germany).

### 4.9. Determination of Glycine Betaine

Glycine betaine (GB) content in the shoots of chia seedlings was estimated using a method previously described in [49] with slight modifications from that described in [50]. Shoot material (0.25 g) from each treatment were ground to a fine powder in liquid nitrogen. The tissue was incubated in tubes containing 20 mL of deionized water for 24 h at 25 °C. The samples were filtered and mixed with 2 N H_2_SO_4_. An aliquot (250 µL) was transferred into a test tube and cooled in ice water for 1 h. Cold potassium iodide-iodine reagent (100 µL) was added, vortexed, and then centrifuged at 1000 × g for 30 min at 4 °C. The sample was incubated for 24 h at 4 °C. The formed periodite crystals were dissolved in 14 mL of 1,2-dichloroethane with gentle agitation at room temperature for 48 h. The absorbance was recorded at 365 nm using a FLUOstar Omega UV-visible spectrophotometer (BMG LabTech GmbH, Ortenberg, Germany).

### 4.10. Statistical Analysis

All experiments described were performed six times independently. For superoxide content, shoot height, shoot fresh weight, shoot dry weight measurements, 30 individual chia seedlings per treatment were analyzed. For all other experiments, 50 chia seedling shoots were homogenized per treatment. For statistical analysis, the one-way analysis of variance (ANOVA) test was used for all data, and means (for six independent experiments) were compared according to the Tukey–Kramer test at 5% level of significance, using GraphPad Prism 5.03 software.

## 5. Conclusions

After summarizing studies on exogenous *p*-CA on plants in a hypothetical model (Figure S2) and incorporating our findings of this study, we conclude that O_2_**^∙^**^−^ plays a crucial role in the downstream signalling mechanism of *p*-CA in chia seedlings. This hypothesis was also observed by [19] where increases in O_2_**^∙^**^−^ led to improved growth of *Brassica napus* seedlings. However, in contrast to [19], our results showed that the regulation of O_2_**^∙^**^−^ content in chia seedlings does not occur via SOD but rather through direct scavenging of O_2_**^∙^**^−^ by *p*-CA [51,52]. Furthermore, we hypothesize that the redox buffering capacity of proline [38] (after direct increase by *p*-CA) specifically with regards to O_2_**^∙^**^−^ plays a major role in synthesis and scavenging of the O_2_**^∙^**^−^ in addition to the direct scavenging capacity of the *p*-CA. This carefully controls the O_2_**^∙^**^−^ levels without triggering an increase in SOD activity. Furthermore, we observed no changes in H_2_O_2_ content and we attribute this to the unchanged SOD activity. We also conclude that the increase in GB by exogenous *p*-CA application led to improved chlorophyll and photosynthetic pigments. This result is supported by findings from [53] which highlighted the role of GB in improving photosynthetic pigments. We hypothesize that *p*-CA improves chia seedling growth via GB and proline activation. Future work should investigate whether the GB pathway occur separately or independently from the proline pathway in chia seedlings treated with *p*-CA.

## Figures and Tables

**Table 1:** Chlorophyll and osmolyte concentration in chia seedling shoots in response to *p*-Coumaric acid. Data represent the means (± SE) of six independent experiments and different letters per row indicate the mean values that are significant different at *p* < 0.05 using the Tukey–Kramer test.

Trait (µg.g^−1^FW)	Control	*p*-CA
Chlorophyll *a*	15.70 ± 0.83^b^	21.30 ± 0.88^a^
Chlorophyll *b*	24.80 ± 1.01^b^	34.40 ± 0.50^a^
Total Chlorophyll	40.50 ± 1.80^b^	55.70 ± 1.38^a^
Carotenoids	1009.70 ± 10.17^b^	1263.30 ± 8.82^a^
Glycine Betaine	6030 ± 233.03^b^	7380 ± 60.02^a^
Proline	1.21 ± 0.02^b^	3.26 ± 0.04^a^

## Supplementary Materials

The following are available online at https://www.mdpi.com/2223-7747/8/12/546/s1, Figure S1. Concentration-dependant germination of chia seedlings (A–B), Method S1: Evaluation of seed germination Figure S2: Schematic model of *p*-CA signalling in chia seedlings.

## Abbreviations

*p*-CA	*p*-coumaric acid
DW	dry weights
FW	fresh weights
GB	glycine betaine
KI	potassium iodide
MDA	malondialdehyde
PUFA	polyunsaturated fatty acids
PVP	polyvinylpyrrolidone
ROS	reactive oxygen species
SH	shoot height
SOD	superoxide dismutase
TBA	thiobarbituric acid
TCA	trichloroacetic acid
