# Supplementary material for: Exogenous p-Coumaric Acid Improves Salvia hispanica L. Seedling Shoot Growth
Source: Plants (Basel). 2019 Nov 26;8(12):546. doi: 10.3390/plants8120546 (PMC6963421; doi:10.3390/plants8120546)
Supplement: Supplementary file 1 [file plants-08-00546-s001.pdf]

Supplementary data

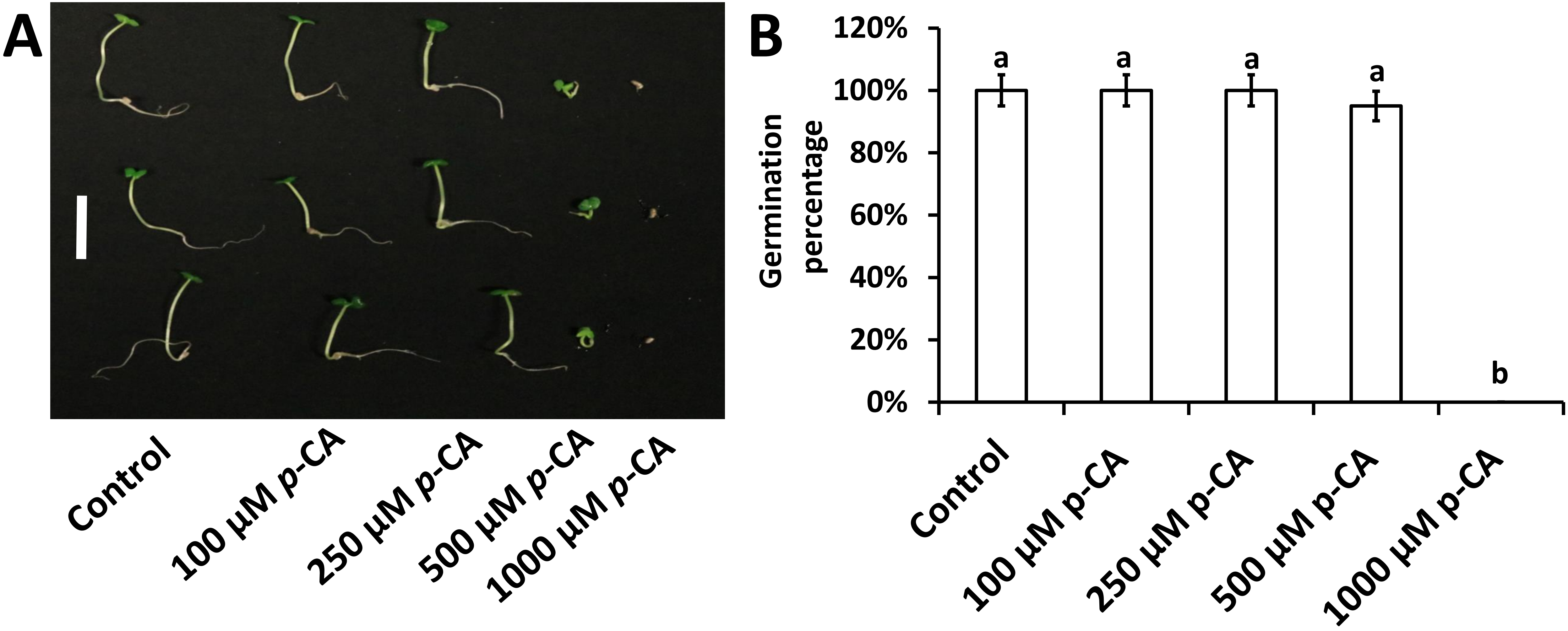

Supplementary Figure S1. Concentration-dependant germination of chia seedlings. The white bar = 2 cm (A). Data represent the means (± SE) of three independent experiments and different letters indicate the mean values that are significantly different at $p < 0.05$ using the Tukey-Kramer test.

Supplementary method:

Evaluation of seed germination

Chia (*Salvia hispanica* L) seeds (50 seeds per treatment) were germinated on half strength Murashige and Skoog (MS) basal media supplemented with different concentrations of *p*-coumaric acid (100 µM, 250 µM, 500 µM and 1000 µM) or without *p*-coumaric acid (control group) in the dark for a period of five days. The seed germination percentage for each treatment (in triplicate) was scored after five days using the following formula:

Seed germination % = (number of seeds germinated)/ (number of seeds per treatment) x 100

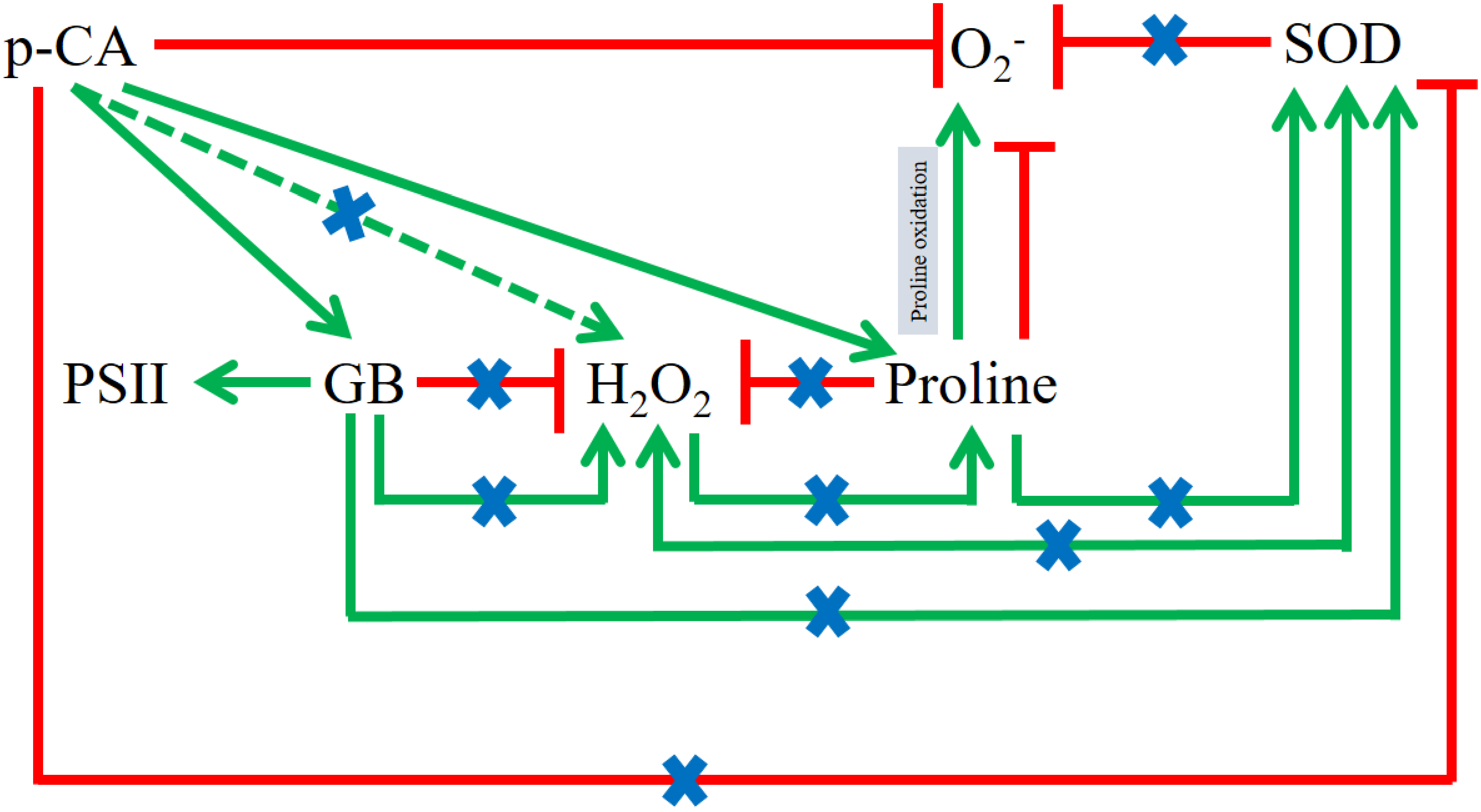

Supplementary Figure S2. Schematic model of *p*-CA signalling in chia seedlings. Inhibition or scavenging (red lines). Activation or increase (green lines). Indirect activation or indirect increase (dashed green line). Did not occur in this study (blue crosses).
